# Assessment of Pain and Inflammation in Domestic Animals Using Infrared Thermography: A Narrative Review

**DOI:** 10.3390/ani13132065

**Published:** 2023-06-22

**Authors:** Alexandra L. Whittaker, Ramon Muns, Dehua Wang, Julio Martínez-Burnes, Ismael Hernández-Ávalos, Alejandro Casas-Alvarado, Adriana Domínguez-Oliva, Daniel Mota-Rojas

**Affiliations:** 1 School of Animal and Veterinary Sciences, Roseworthy Campus, University of Adelaide, Roseworthy, SA 5116, Australia; 2 Agri-Food and Biosciences Institute, Hillsborough, Co Down BT 26 6DR, Northern Ireland, UK; 3 School of Life Sciences, Shandong University, Qingdao 266237, China; 4 Facultad de Medicina Veterinaria y Zootecnia, Universidad Autónoma de Tamaulipas, Victoria City 87000, Mexico; 5 Clinical Pharmacology and Veterinary Anesthesia, Facultad de Estudios Superiores Cuautitlán, Universidad Nacional Autónoma de México (UNAM), Cuautitlán 54714, Mexico; 6 Neurophysiology, Behaviour and Animal Welfare Assessment, DPAA, Xochimilco Campus, Universidad Autónoma Metropolitana, Mexico City 04960, Mexico

**Keywords:** nociception, inflammatory response, invasive procedures, surgery, analgesia, castration, teeth clipping, infrared thermography (IRT)

## Abstract

**Simple Summary:**

Current acute pain assessment protocols in domestic animals require the use of a suite of methods to evaluate the multidimensional components of pain. Thermal imaging is a method that assesses the physiological response, via heat production, to a noxious stimulus. This may correlate with the affective experience of acute pain. Since the use of the method as a pain assessment tool is still in its relative infancy and has yet to be fully evaluated empirically, the present review brings together current knowledge on the use of infrared thermography to evaluate acute pain in animals. We also propose future directions in this area of research.

**Abstract:**

Pain assessment in domestic animals has gained importance in recent years due to the recognition of the physiological, behavioral, and endocrine consequences of acute pain on animal production, welfare, and animal model validity. Current approaches to identifying acute pain mainly rely on behavioral-based scales, quantifying pain-related biomarkers, and the use of devices monitoring sympathetic activity. Infrared thermography is an alternative that could be used to correlate the changes in the superficial temperature with other tools and thus be an additional or alternate acute pain assessment marker. Moreover, its non-invasiveness and the objective nature of its readout make it potentially very valuable. However, at the current time, it is not in widespread use as an assessment strategy. The present review discusses scientific evidence for infrared thermography as a tool to evaluate pain, limiting its use to monitor acute pain in pathological processes and invasive procedures, as well as its use for perioperative monitoring in domestic animals.

## 1. Introduction

Pain is a multidimensional sensory and emotional experience that it is now accepted that animals can perceive, regardless of their inability to verbalize the experience, as discussed in the latest definition proposed by the International Association for the Study of Pain [1]. The current approach to pain management in animals is through categorizing physiological and behavioral changes in an attempt to provide timely treatment and reduce welfare impact [2]. However, behavioral-based pain scales, one of the most common strategies to assess this negative state, are often subjective and dependent on the observer. This impacts the reliability and confidence we can have in the findings [3,4].

Infrared thermography (IRT) has been proposed as an objective method to evaluate acute pain, with a basis in the physiological response during pain perception [5,6]. Temperature variations can be measured using IRT after a nociceptive stimulus due to the activation of the nervous system and its vasomotor consequences, inducing an inflammatory process and an increase or decrease in heat radiation [7,8]. Therefore, theoretically, IRT can assist in monitoring the physiological function and pain status of animal patients. Moreover, the non-invasiveness of IRT makes it a tool that can be used in animals with complex management [9].

However, there is still controversy as to whether this temperature variation response is associated with pain level and if it is reliable as a guide to adjusting analgesic treatment during invasive procedures in animals. Likewise, selecting the anatomical region or thermal window is essential since the nociceptive process induces peripheral vasoconstriction or vasodilation depending on the evaluated region, influencing the amount of heat radiation [10,11]. Research on IRT is still limited; thus, this review will bring together current research on IRT applied to evaluate acute pain in domestic animals, discuss gaps in knowledge, and propose avenues for future investigation restricted to acute pain monitoring.

## 2. Search Methodology

For the present review, a literature search was performed in databases such as Web of Science, Scopus, and PubMed. The following keywords were used: “domestic animal infrared thermography”, “animals inflammatory process”, “animal surgery”, “acute painful surgical procedures in animals” and “neurobiology of acute pain in animals”. Inclusion criteria were that there was full-text access, they were written in English or Spanish, and there was a discussion of IRT in the context of evaluating pain during inflammatory diseases or surgical procedures. Likewise, those articles mentioning the neurobiology of pain and its physiological changes were used to describe the physiological basis of IRT. Articles were excluded if they used IRT to assess body temperature but did not establish any association with acute pain. In total, 147 references were cited in the present review.

## 3. Neurobiology of Pain and Its Association with the Superficial Thermal Response

Pain presence is highly relevant to the health and well-being status of animals. The pain experience leads to behavioral and physiological consequences which differ in magnitude based on intensity [3,12,13]. An understanding of the neurobiological process underlying pain perception is necessary to understand the relationship between pain and thermal response.

The neurobiological process of pain consists of five phases, which, according to authors such as Bell [14] and Lamont et al. [15], are (1) transduction, (2) transmission, (3) modulation, (4) projection, and (5) perception [16]. Transduction is the transformation of mechanical, thermal, and chemical stimuli to an electrical impulse due to nociceptors present in peripheral tissues [17,18]. Transient receptor potential (TRP), particularly TRPV1, TRPV2, TRPV3, TRPV4, TRPM8, and TRPA1, participates in this phase by transducing noxious stimuli [17,19,20]. These receptors alter the action potential when they detect temperature disturbances [21]. For example, Kozyreva et al. [22] evaluated anesthetized rats receiving two cold thermal stimuli types. They found that TRPM8 activation by cold, with the use of menthol as an agonist of this receptor, increased oxygen consumption and induced peripheral vasoconstriction to shift the heat and maintain core temperature. The use of in vitro models has shown that activating TRPV1 channels in response to hot thermal stimuli increase the entrance of K^+^ or Na^+^ ions to promote energy expenditure and thermogenesis by decreasing visceral fat accumulation [23,24]. These examples show that TRPs initiate the response to thermal disturbances and painful sensations that are subsequently transmitted by type Aδ and C nociceptive fibers [25].

The activation of nociceptors during the transduction and transmission of a painful stimulus triggers different thermal responses that can be local (inflammation) or systemic (autonomic response). To achieve this, the modulation of the signal in the dorsal horn of the spinal cord suppresses or enhances the projection and perception of pain in cerebral structures [14,26,27]. Different brain regions activate due to pain, including the primary and secondary somatosensory cortices, cingulate cortex, insular cortex, and amygdala [28,29].

The central nervous system (CNS) processing contributes to the thermal response since the nerve signal is transmitted through the spinothalamic tract, resulting in the neurosecretion of catecholamines (epinephrine and norepinephrine). This also occurs in the locus coeruleus and the thalamus, transmitting nociceptive information to the somatosensory cortex, hypothalamus, and hippocampus [30,31]. Through this mechanism, pain induces the activation of the hypothalamic-pituitary-adrenal axis, the sympathetic nervous system, and, consequently, the release of glucocorticoids [32,33]. On the other hand, activation of the sympathetic-adrenomedullary system alters cardiovascular function due to catecholamine secretion by activating adrenoceptors in the vascular endothelium [34,35]. This event causes hypertension, tachycardia, tachypnea, and changes in peripheral and central temperature [35]. Redaelli et al. [36] used IRT to monitor rodents with induced spinal cord injury. In these animals, the temperature of the interscapular region, where brown adipose tissue is located, increased by 5 °C. This effect could be explained by thermogenesis, and the increase in the availability of energy resources is due to gluconeogenesis, lipolysis, and proteolysis [37]. Therefore, these mechanisms may clarify the value of IRT as an assistance tool for pain recognition [11,38].

**Figure 1:**
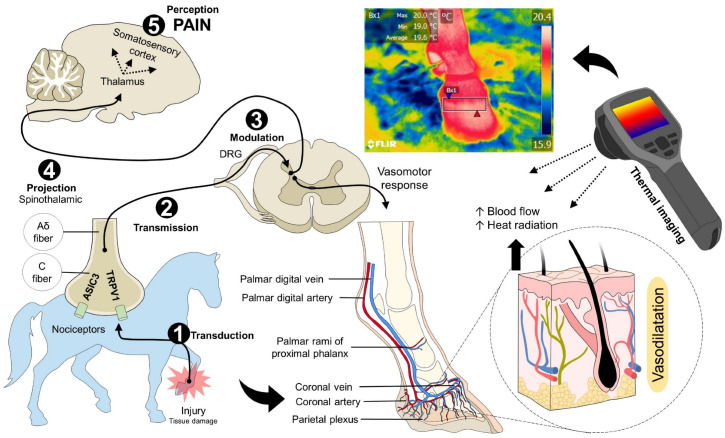
Nociceptive pathway during laminitis. The inflammatory process and tissue injury caused by laminitis trigger the first phase of nociception. (1) Transduction. The noxious stimulus is recognized and transformed into an electrical signal by peripheral nerves (Aδ and C fibers), known as nociceptors. In these free nerve endings, receptors such as ASIC3 or TRPV1, among others, are activated to create action potentials that will be transmitted to the DRG of the spinal cord. (2) Transmission. Through Aδ and C fibers, the noxious input is transmitted to the spinal cord, to synapse with second-order neurons in the gray matter of the structure. (3) Modulation. Once the signal reaches the spinal cord, spinal interneurons are responsible for projecting or modulating the signal by releasing inhibitory or excitatory neurotransmitters. (4) Projection. Through the spinothalamic tract, the electrical signal reaches superior centers in the brain, mainly the thalamus. (5) Perception. From the thalamus, third-order neurons project to the somatosensory cortex, where the conscious recognition of pain is developed. Due to the interaction of the thalamus with other regions such as the hypothalamus, pain activates sympathetic centers, which causes physiological, endocrine, and behavioral responses. The vasomotor response, occurring as a result of pain and inflammation, causes vasodilation in the injured site to promote immune cells invasion to the injury site, with consequent healing. The increase in blood flow in the region also increases the amount of radiated heat from the skin. This element is captured by thermal cameras, helping to identify an inflammatory process in animals. ASIC: acid-sensing ion channel; DRG: dorsal root ganglion; TRPV1: transient potential receptor vanilloid 1.

Finally, when tissue injury occurs, an inflammatory process arises due to the release of pro-inflammatory substances such as cytokines, interleukins, prostaglandin, and ions, which can alter the action of the nociceptors, thus facilitating the activation of TRP channels, a phenomenon known as peripheral sensitization [39,40]. Local heat is a consequence of the vasodilation caused by pro-inflammatory mediators, altering the amount of dissipated heat [41]. Through these mechanisms, it has been suggested that IRT can be used to recognize processes such as laminitis, mastitis, or tendon injuries [5,42]. This evidence strongly suggests that the neurobiological process of pain is related to the thermal response, as shown in Figure 1, and this reaction can be identified using IRT.

## 4. Inflammatory Responses and Pain during the Pathological Process and Its Relation to Thermal Changes

Inflammatory responses associated with acute pain result from sudden or constant exposure to noxious stimuli and the interaction between pro- and anti-inflammatory mediators [43]. They serve as a mechanism for recovery from injury, trauma, sepsis, or infections. Nonetheless, mismanagement of acute inflammation can gradually lead to chronic disorders and persistent pain [44,45]. The labels of acute or chronic are generally based on a time construct, which typically ranges from a few days (acute) to weeks (chronic). There may also be differences in pain intensity associated with these different timelines; for example, chronic pain may be of a lesser intensity but prolonged.

**Figure 2:**
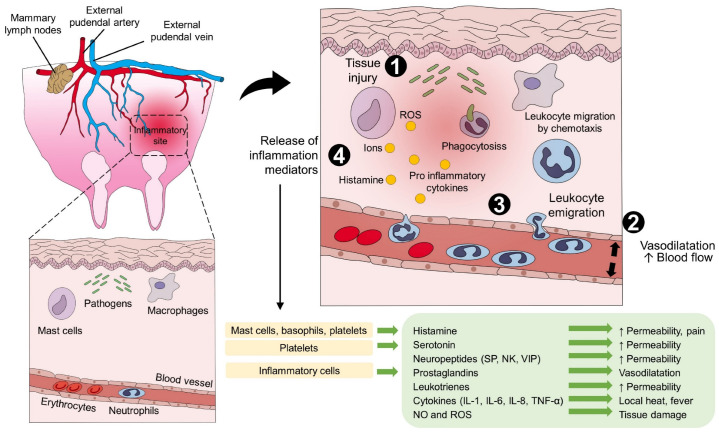
The local inflammatory response in the udder of a patient with mastitis. (1) The presence of a tissue injury or pathogens initiates inflammation by releasing local chemical compounds. (2) First-line mediators such as histamine, NO, ROS, and ions induce vasodilation to increase the permeability of the blood vessels and blood flow. (3) Leukocyte activation, emigration, and migration cause the presence and degranulation of neutrophils and other inflammatory cells into the injury site. (4) Release of pro-inflammatory substances such as prostaglandins, leukotrienes, cytokines, and neuropeptides, among others, exacerbates the inflammatory response to promote pathogens’ phagocytosis, and healing IL: interleukin; NK: neurokinin; NO: nitric oxide; SP: substance P; VIP: vasoactive intestinal polypeptide.

Pain, as one of the cardinal signs of inflammation, is accompanied by an increase in core and surface temperature at the injury site—an element that can be monitored with IRT—due to the interaction between pro-inflammatory mediators and nociceptors. At the inflammatory site, prostaglandins, prostacyclin, interleukins (IL), leukotrienes, cytokines, histamine, serotonin, bradykinin, and alpha tumor necrosis factor (TNF-α), among others, induce vasodilatation and increase blow flow and permeability of blood vessels, emigration of leukocytes, migration by chemotaxis to the injury site, and the release of chemical mediators, causing signs such as redness, swelling, pain, loss of function, and local heat (Figure 2) [46,47,48,49,50].

**Figure 3:**
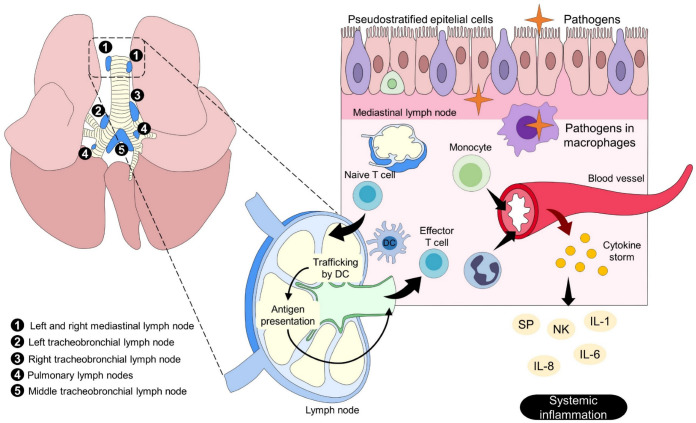
The systemic inflammatory response in pneumonia. Infection of the airway epithelium by pathogens leads to an inflammatory response where the mediastinal lymph node has a crucial role by participating in trafficking by DC, known as the take up of pathogens to the lymph node. Within the lymph node, antigen presentation takes place on naïve T cells to release effector T cells and induce the adaptative response of the organism. Degranulation of monocyte and polymorphic cells into the bloodstream causes a cytokine storm (SP, NK, IL), a reaction that promotes systemic inflammation and clinical signs. DC: dendritic cells; IL: interleukin; NK: neurokinin; SP: substance P.

Pain during an acute inflammatory process can be maintained by mast cells releasing histamine [51], bradykinin, and prostaglandins [52]. Pro-inflammatory substances such as Cytokines as IL-1β, IL-6, and TNF-α act on Toll-like receptors and TNF receptors [53], triggering various systemic changes, ranging from blood parameter alteration, organ failure, and even death, such as in the case of pneumonia in dogs (Figure 3) [54,55,56,57]. In the respiratory system, aspirated pathogens are removed by the mucociliary system and eliminated by alveolar macrophages and neutrophils. Alternatively, those reaching the lung by blood circulation are attacked by intravascular macrophages. In addition, phagocytosed pathogens by macrophages and dendritic cells are conducted to lymphoid tissues, allowing B and T Lymphocytes to contact pathogens and contributing to the humoral and cellular immune response. Antigen recognition in the lymphoid tissue leads to T cell proliferation and differentiation; hence, differentiated effector and memory T cells enter the circulation, migrate to the antigen site, activate macrophages, and kill ingested pathogens. However, activated cells in the bloodstream release cytokines producing cytokine storm and systemic inflammation [58].

Recently, IRT has been implemented in veterinary medicine. Infrared cameras can be divided into two types: cooled and uncooled array detectors. Cooled cameras have limited application for clinical use because they need to cool down before taking the image. Therefore, the process must be organized in advance [59]. On the other hand, uncooled thermal cameras use pyroelectric or ferroelectric materials for infrared radiation detection and pixel formation. Uncooled detectors constantly calibrate according to ambient temperature, being suitable for clinical application [60,61,62,63]. However, uncooled chamber detectors cannot provide a fixed absolute temperature due to constant calibration. Therefore, measuring the absolute temperature of the subject is not possible without compensatory programs for the internal temperature of the detector [64]. Moreover, IRT cameras also differ according to the number of lenses. Research-grade thermal cameras are characterized by three lenses that determine the focal length [65]. This is relevant because the capacity of the camera to detect thermal variability is higher in smaller spot-size lenses [65].

**Figure 4:**
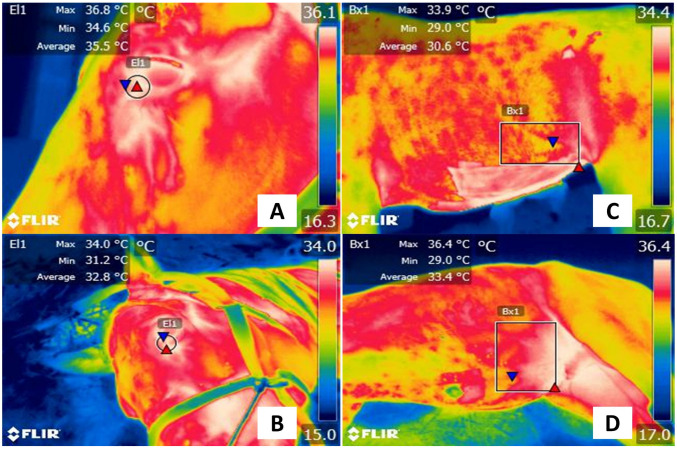
Superficial thermal response associated with acute pain in a horse with colic. (**A**,**C**). Healthy female quarter-horse patient. The maximum temperature in the lacrimal caruncle (El1) was 36.8 °C (red triangle), and the minimum was 34.6 °C (blue triangle). In the caudal abdominal region (Bx1), the surface temperature presented a maximum temperature of 33.9 °C (red triangle) and a minimum of 29 °C (blue triangle). (**B**) Thoroughbred female equine with acute pain associated with colic. It is observed that the maximum (red triangle) and minimum (blue triangle) temperatures of the lacrimal caruncle (El1) decreased by 2.8 °C and 3.4 °C, respectively, compared to the healthy horse. (**D**) Contrary to the thermal response in the lacrimal caruncle, in the caudal abdominal region (Bx1) of the sick animal, the maximum surface temperature increased by 2.5 °C. The thermal images were taken by the authors of the present review. Radiometric images were obtained using a T1020 FLIR thermal camera. Image resolution 1024 × 768; up to 3.1 MP with UltraMax. FLIR Systems, Inc. Wilsonville, OR, USA.

These cameras have been used in animals to study diseases such as neoplasia [66], musculoskeletal disorders [42], vascular, metabolic disorders, and infectious conditions associated with fever [34,67]. For example, in equines, infectious colic accompanied by fever and pain has been assessed in preliminary studies. Figure 4 shows the thermal differences between a healthy mare and a mare with acute colic. In the sick horse, the inflammatory response arises from the presence of cytokines and vasoactive amines, causing vasomotor changes and altering the radiated heat detected by IRT. Contrary reactions were also found in these patients, with a decrease in ocular temperature, due to vasoconstriction, and an increase in caudal abdominal temperature due to vasodilation and cytokine release. In equines, common disorders such as colic are characterized by acute pain and local inflammation of the intestinal mucosa. Due to this event, as shown in Figure 3, IRT could help to identify these vascular changes. However, limited case reports have been published about this pathology and its association with IRT, limiting its use [68]. Subsequent studies by Schaefer et al. [69,70] have suggested that implementing IRT can help to diagnose febrile states in domestic animals due to infectious conditions. According to these same authors, in domestic cattle with Bovine Respiratory Disease Complex, they show an increase in maximum surface temperature due to an increase in neutrophils/lymphocytes [71]. Other studies reported an IRT efficiency in recognizing this disease between 71–80% when compared to standard diagnostic methods [72]. This would reinforce the recommendation of using IRT as an assistance tool for recognizing infectious states in farm animals [73,74,75,76], in addition to its application in humans to identify febrile patients in airports during the COVID-19 pandemic [77,78].

**Figure 5:**
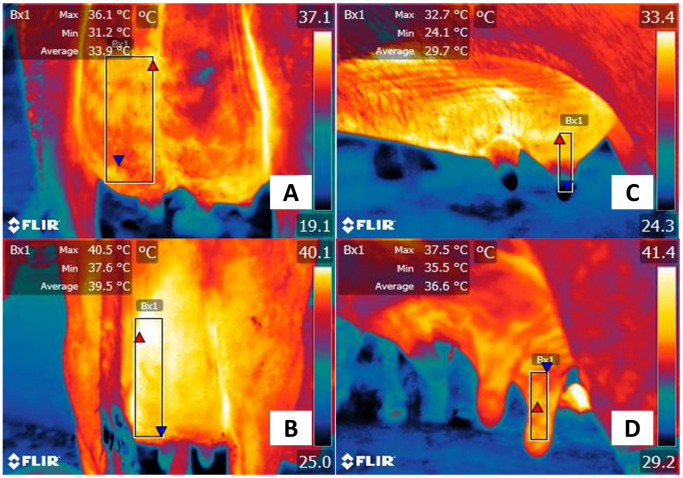
Thermographic evaluation of the thermal response of the mammary gland in dairy cows and buffaloes. (**A**) Healthy udder of a Holstein bovine. The surface temperature of the caudal quarter (Bx1) of the udder shows a maximum temperature of 36.1 °C (red triangle) and a minimum value of 31.2 °C (blue triangle). (**B**) Udder of a Holstein cow with mastitis. The maximum and minimum temperature of the caudal quarter (Bx1) is 4.4 °C and 6.4 °C higher, respectively. (**C**) Healthy udder in a Murrah water buffalo. The surface temperature of the healthy lateral caudal quarter (Bx1) recorded a maximum temperature of 32.7 °C (red triangle) and a minimum of 24.1 °C (blue triangle). (**D**) Udder with mastitis in dairy buffalo. Compared with the thermal response of the healthy udder, the temperature of the caudal quarter (Bx1) of the udder is 4.8 °C higher at the maximum temperature (red triangle) and 11.4 °C of the minimum temperature (blue triangle) compared to the temperature of the healthy udder. Infectious agents such as *Escherichia coli* or *Staphylococcus* sp. can trigger the release of several pro-inflammatory cytokines that cause vasodilation and increase heat radiation associated with acute pain. The thermal images were taken by the authors of the present review. Radiometric images were obtained using a T1020 FLIR thermal camera. Image resolution 1024 × 768; up to 3.1 MP with UltraMax. FLIR Systems, Inc. Wilsonville, OR, USA.

Mastitis is a prevalent disease in dairy cattle [79,80,81] that affects animals’ health and causes economic loss due to a reduced milk yield [82,83]. It is a disease not always accompanied by clinical signs, although local inflammation and pain are commonly present in the udder. Therefore, the ability to diagnose this condition by IRT based on udder surface temperature would be valuable (Figure 5) [71]. Machado et al. [84] evaluated subclinical mastitis in 38 cows using thermal imaging of the udder. The authors observed a high correlation between the udder temperature and the somatic cell count. This relation was predominant in the posterior quarter of the udder, where the recorded temperature in the right quarter was 40.6 °C, while the left side recorded 39.8 °C, with correlation values of R2 = 0.97 and R2 = 0.88, respectively. This illustrates that the technique may not only be useful for binary evaluations based on the presence or absence of disease but may be able to detect severity.

In another study, Zaninelli et al. [85] evaluated the udder health status of 310 dairy cows through somatic cell counts and IRT in 3 farms under field conditions. The authors observed that sick animals presented a maximum temperature of 36.08 ± 0.22 °C and a somatic cell count of 930.81 ± 96.58 × 10^3^. In contrast, healthy cows had a maximum temperature of 35.79 ± 0.15 °C and a somatic cell count of 592.38 ± 71.40 × 10^3^, finding a correlation between both variables. Thermographic research in infectious processes has highlighted that activation of the immune system can cause substances such as IL-1, IL6, TNF-α, and PGE2 to cross the blood-brain barrier and, therefore, act on the anterior preoptic area of the hypothalamus, resulting in heat production and increasing the radiated energy, being able to differentiate with IRT the sick animal from the healthy ones [86,87].

**Figure 6:**
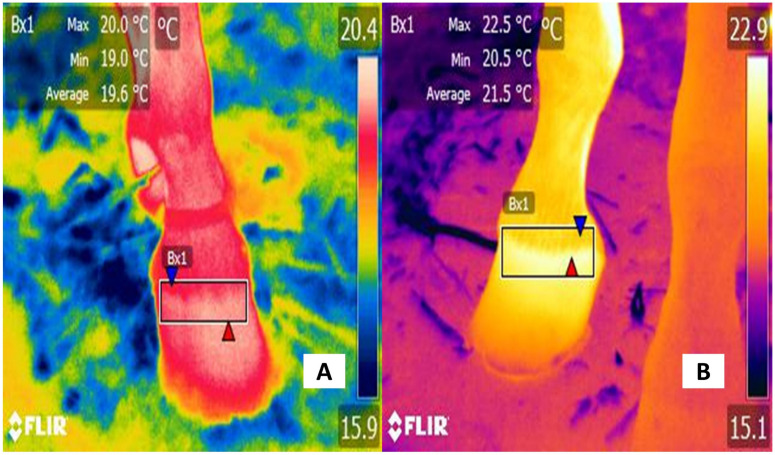
Thermographic evaluation of hoof inflammation due to laminitis. (**A**) Healthy hoof. The hoof of a quarter-horse male with no reported pathologies. A maximum temperature of 20 °C (red triangle) and a minimum of 19 °C (blue triangle) can be observed on the surface of the coronet band (Bx1). (**B**) Hoof with laminitis. The thermal response of the coronet band (Bx1) of a female horse of the Azteca breed recorded increases by 2.5 °C and 1.5 °C at the maximum (red triangle) and minimum temperature (blue triangle). This may be due to the invasion of infectious agents during laminitis, which can trigger the release of interleukin-1, interleukin-10, histamine, and prostaglandin F2 alpha, causing an increase of heat in the inflamed area. The thermal images were taken by the authors of the present review.

Hoof and locomotion disorders, characterized by lameness (Figure 6), are other examples of chronic inflammatory conditions that have been evaluated with IRT. Weimer et al. [88] applied IRT to study bacterial chondronecrosis with osteomyelitis (BCO) secondary to laminitis in chickens. In the study, chickens with lameness had severe lesions caused by BCO diagnosed macroscopically. Lower temperatures were recorded in the hock joint, shank, tarsometatarsus, foot leg, and phalangeal regions. However, the investigators concluded that IRT was more accurate in identifying an increase in leg temperature in subclinical chondronecrosis lesions rather than a temperature drop in clinical lesions.

Fever is another sign of infection that IRT may be able to identify whilst minimizing animal handling [69,89]. IRT could help in the early diagnosis of diseases with generalized subcutaneous edema, nasal discharge, oral lesions, cyanotic tongue, dyspnea, and lameness [90,91]. This could be extremely valuable in the case of notifiable diseases such as bluetongue allowing early detection of fever and swift management and eradication. Pérez de Diego et al. [92] implemented IRT in 9-month-old male Merino sheep experimentally infected with bluetongue virus [93,94]. The surface temperature of the eye and the surrounding skin was taken, with blood samples being collected on the second-day post-inoculation. A moderate correlation between rectal temperature and IRT (r = 0.504) was found, although IRT registered lower values than rectal temperature. However, the difference was only 1.46 ± 0.05 °C, which is unlikely to be of clinical significance, i.e., IRT is a good proxy for rectal temperature. Another condition that can result in systemic inflammation is autoimmune encephalomyelitis. This disease is characterized by excessive cytokine production associated with fever, fatigue, sleep disturbances, myalgia, and cognitive disorders [95]. Faraji et al. [96] reported a high susceptibility to autoimmune encephalomyelitis among female mice. Females not only presented with tail weakness, paralysis, and increased levels of pro and anti-inflammatory cytokines but also had higher dorsal temperatures than males (females vs. males; 37.72 ± 1.63 °C vs. 30.61 ± 1.59 °C; t18 = 3.06, *p* < 0.01).

These studies demonstrate that IRT can detect relevant differences between healthy and sick animals and that it can serve as an additional tool to detect inflammation, infectious diseases, and pathological processes or to determine the severity of the disease when a greater thermal variation is observed.

## 5. IRT as an Assistance Tool in Invasive and Surgical Procedures

### 5.1. Surgical Procedures

Several husbandry interventions (e.g., disbudding, castration, teeth clipping, tail docking, among others) are known to cause pain in animals. This pain is exacerbated by inappropriate analgesic or anesthetic protocols [97]. A survey by Fajt et al. [98] reported that only 30.5–33.9% of calves under six months old receive analgesics during castration and 62.5–68.1% during dehorning. In piglets, a survey in 24 European countries found that 54% of animals are castrated without anesthesia and analgesia [99]. Similarly, only 42% of sheep producers in Australia use pain relief for castration and tail docking [100].

The lack of analgesia causes physiological and behavioral responses associated with acute pain perception. These responses have been monitored through behavior-based scales, pain biomarkers (e.g., cortisol, substance P), cardiorespiratory variables (heart rate and respiratory rate), and productive performance (body weight, daily food intake) [101]. Some studies have implemented IRT to assess the presence/absence of pain and the pharmacological efficacy of compounds proposed within analgesic protocols to promote welfare during these invasive procedures [102]. The following discusses these investigations.

Castration is a common management technique used across species to control breeding. The procedure consists of either the physical removal or chemical inactivation of the testicles. These events cause inflammation and pain due to the manipulation of soft tissues [103]. The inflammatory response generated has been studied in tomcats using superficial testicular IRT to determine the clinical viability of chemical castration—a combination of 20% CaCl_2_ with 0.5% dimethyl sulfoxide—at 18-time points. Paranzini et al. [104] showed that this treatment caused a minimal local inflammatory reaction with only a decrease in IRT values 10 min after the injection due to temporary vasoconstriction. The unusual results with the opposite of the expected response may be reconciled since the cats demonstrated a normal behavioral repertoire, suggesting a lack of pain.

Unlike dogs and cats, farm animals rarely receive analgesia or anesthesia during castration. Reasons for this issue include that, in many countries, there are no approved products for pain management during surgical castration in species that will enter the food chain. If a drug can be used, it must be administered by aveterinarian [105]. This has led to a large body of research studying animal pain during or after on-farm procedures and the testing of alternative procedures or alternative agents to mitigate pain. Some of these have employed IRT. For example, CO_2_ surgical lasers for piglet castration were tested, and IRT of the surgical wound was used to show that, in comparison to scalpel-castrated piglets, temperatures were significantly lower [106]. However, despite this finding, behavioral evaluations showed that laser-castrated animals displayed more pain-related behaviors (e.g., scratching, stiffness, trembling, and tail wagging), and greater tissue damage around the incision site was observed. The latter may explain the unexpected finding since damage can alter vascularization in the zone, influencing IRT reading. This finding highlights the need for further research on IRT and the factors influencing its response prior to widespread use.

Aside from castration, other invasive procedures, such as otectomy and gastrotomy, have been evaluated using the superficial temperature of the surgical wound. Saidu et al. [107] evaluated the temperature of surgical wounds in indigenous dogs subjected to castration, otectomy, and gastrotomy, registering increases within the first 12 h post-surgery (+2.34 °C, +0.58 °C, and +0.56 °C, respectively), and, at 48 h, the temperature in the first two groups was higher than those undergoing gastrotomy. Accordingly, the authors concluded that increased values could be associated with pain and inflammatory responses, an effect that was not observed in gastrotomy. However, the lack of significant changes in gastrotomy does not necessarily imply that this procedure is non-painful since IRT reading depends on blood supply from the skin. It is plausible that the lack of blood supply to the linea alba, where the incision would have been made, may have altered the results in this scenario.

### 5.2. Efficacy of Analgesics

IRT has also been used to evaluate the administration of drugs for pain management. In calves, Stewart et al. [108] evaluated the effect of surgical castration using maximum eye temperature and physiological variables such as heart rate variability, heart rate, and concentrations of cortisol, epinephrine, and norepinephrine. When comparing the effect of the surgical castration alone, a surgical procedure with local analgesia, and sham handling as the control group, eye temperature increased significantly in the patient’s undergoing surgery, regardless of the analgesia. This effect also corresponded with cortisol increases. The authors concluded that IRT could assess the activation of the autonomous nervous system (ANS) after the perception of acute pain since both the endocrine and physiological responses were associated with increased superficial temperature. The efficacy of topical analgesics was assessed in Holstein calves receiving 3.33 mg/kg of transdermal flunixin meglumine applied to the dorsal midline during castration. Treated animals registered the highest ocular temperatures compared to placebo and previously castrated animals (35.4 °C, 34.5 °C, and 34.3 °C, respectively). Although cortisol concentrations were significantly lower in the treated animals, substance P concentrations were similar between all experimental groups. The authors concluded based on the results that transdermal flunixin had little analgesic effect [109]. A similar result was observed by Graves et al. [110] in a study on castrated mix breed goats receiving the same analgesic protocol. Scrotal IRT, heart rate, respiratory rate, and rectal temperature were measured with the finding that there was no difference between flunixin and placebo-treated groups for the physiological parameters measured However, there was some evidence of a flunixin effect as food intake was improved in the analgesic group. The authors reconciled that the analgesic may have had some effect but possibly needed dose refinement to show the change in physiological parameters expected. 

Electroencephalography and ocular IRT were used to assess pain in another castration study on merino ram lambs [111]. Animals were allocated to [111] groups of castration with meloxicam and lignocaine, castration untreated and sham [111]. At 1 h post-surgery, untreated animals and those that received the drug recorded increases in ocular temperature of approximately 0.3 °C (basal = 38.6 °C vs. 1 h post-surgery = 38.8 °C). In the groups receiving meloxicam and lignocaine, the temperature increased 0.6 °C at 4 h post-surgery (basal = 38.6 °C vs. 4 h post-surgery = 39.2 °C), which was likely a consequence of waning analgesia. Given that there were abnormal lying, standing, and walking behavioral responses, it was suggested that the analgesic protocol was not efficacious [111]. In contrast, successful analgesic treatment in piglets via milk was reported by Bates et al. [112], who administered meloxicam orally to Yorkshire X Landrace sows—after farrowing—to test the transfer of the drug through milk. On post-farrowing day five, gilts and piglets were subjected to castration, tail docking, and iron injection. Treated animals had lower cortisol concentrations with no differences in mean plasma substance P levels. IRT at the lower cranial region (including cranial, left, and right ear, and snout temperature) was lower in control piglets (sham handling) than in treated animals, concluding that animals receiving meloxicam maintained a consistent temperature before and after the procedures, and the temperature values were similar to those registered in the control group.

An often-cited justification for the lack of analgesia provision to newborn or very young farm animals is the belief that their nociceptive pathways are not fully developed and are incapable of perceiving pain in the same way as mature animals [101,113]. Nonetheless, studies with animals of different ages dispute this assertion. Bergamasco et al. [97] evaluated unmitigated surgical castration in Holstein calves at different ages (six weeks, three, and six months) and after exposure to a simulated castration. In general, average surface eye temperature was higher in the castration groups but in both groups, a significant decrease was recorded. This change can be attributed to sympathetically mediated vasoconstriction and an adaptative response of the parasympathetic system to painful stimuli. However, there was an effect of age, where calves of six weeks had lower temperatures than older calves. Additionally, younger animals had lower cortisol concentrations, with peak values returning faster to baseline (within 50 min post-surgery) than in older animals (120 min). This information is critical since it does not imply that younger animals are not in pain and should be withheld analgesia but that analgesic protocols must consider age to guide appropriate drug choice and dose selection.

The effect of different castration techniques on pain generation has also been studied with IRT. In Holstein claves, castration by banding, cut-and-clamp, and cut-and-pull, plus the influence of bull calves’ age was evaluated using ocular IRT and electroencephalography, electrodermal activity, and pain biomarkers (cortisol and substance P). The electroencephalogram recorded desynchronization in all treatments, representing general arousal but greater desynchronization in older calves, with higher cortisol concentrations and increased electrodermal activity. Maximum eye temperature had an apparent increase after treatment in all groups and ages, but average IRT temperature was affected by age, where bulls of 6 months recorded the highest temperatures [114]. Similarly, in beef cattle, castration by band and knife in animals of 3 different ages (1 week, 2 months, and 4 months old) was assessed by IRT in the scrotal area, together with salivary cortisol, substance P, haptoglobin concentrations, and activity levels [115]. This study concluded that pain treatment should be provided, particularly to calves older than 2 months old, where band castration registered the highest temperatures (37.2 °C), and inflammation was maintained for up to 14 days with swelling for up to 35 days. These indicators could suggest a chronic pain response and point to the potential utility of IRT to assess this more long-lived response [115]. Both examples show a response associated with the activation of the ANS and biomarker release, such as nitric oxide. Taken together, these findings suggest that castration can be performed at an early age, but with consideration for hyperalgesia effects and the importance of analgesic protocols in all groups of animals [114].

**Figure 7:**
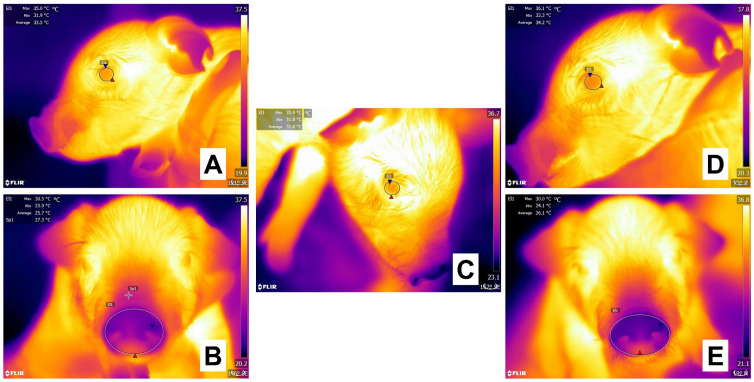
Evaluation of the thermal response of 1-day-old piglets during tail docking with side-cutter pliers. (**A**,**B**) Before tail docking, the ocular surface (**A**, El1) of the piglet registered a maximum temperature (red triangle) of 35.0 °C, while the nasal window (**B**, El1) had a maximum temperature (red triangle) of 30.5 °C. (**C**). During the procedure, the ocular surface window increased its maximum temperature by 0.9 °C. (**C**,**D**) When comparing basal values with the surface temperatures taken immediately after tail docking, the maximum temperature of the piglet at the ocular window (**D**, El1) increased by 1.1 °C. At the same time, the nasal region (**E**, El1) decreased by 0.5 °C. This ambivalent reaction could be a result of the HPA axis increasing blood flow to important organs such as the eye and limiting it to a peripheral region such as the piglet’s nose. Sp1: default focal point of the software. The thermal images were taken by the authors of the present review. Radiometric images were obtained using a T1020 FLIR thermal camera. Image resolution 1024 × 768; up to 3.1 MP with UltraMax. FLIR Systems, Inc. Wilsonville, OR, USA.

IRT has also been used to monitor routine procedures such as tail docking, teeth clipping, and castration in piglets to assess the physiological changes and the analgesic efficacy of different compounds. Tail docking is used to prevent tail-biting behavior in piglets [116]. Figure 7 shows the sequence of thermal images taken in 1-day-old piglets (White Large x Landrace) before, during, and after tail docking with side-cutter pliers. In general, an increase in temperature on the ocular surface and a decrease in the nasal region are observed, a reaction that could be attributed to acute pain perception. In the case of dairy cattle, the presence of chronic pain due to tail docking and increased sensitivity to heat and cold was shown by Eicher et al. [117] through thermal imaging of the tail. When comparing docked and non-docked animals, after the sensitivity test, tails from docked animals remained 1.43 °C warmer, showing the increasing sensitivity as a result of the procedure. This is similar to the phantom limb effect in humans. These studies show that IRT could also be applied to monitor acute and chronic pain.

Dehorning, another practice commonly performed on farm animals, was studied by Van der Saag et al. [118] in Hereford calves. The analgesic effect of buccal meloxicam and local anesthetics was studied using IRT on dehorning wounds, in the first seven days after dehorning. In general, there was no effect of treatment on behavioral and thermographic parameters. However, temperature increases were time-dependent with increases from day 1 (38.83 ± 0.42 °C) to days 3 and 7 (40.43 ± 0.35 °C, 40.30 ± 0.40 °C, respectively). In another study in dairy calves, cautery disbudding was associated with increased nociception as shown by ocular IRT, plasma cortisol, substance P concentrations, and pressure algometry. When comparing animals receiving a single oral dose of firocoxib (0.5 mg/kg) and placebo after a cornual block, the animals receiving placebo had higher cortisol concentrations and heart rates. However, no significant differences were found in ocular IRT between groups (both groups recorded 37.7 ± 0.1 °C), although a decrease by 0.12 °C and 0.15 °C was reported between 2 and 4 h after disbudding, respectively [119]. These results suggest that firocoxib dose might reduce nociception, particularly during the first 24 h post procedure, but IRT alone might not be suitable to determine its analgesic efficacy.

**Figure 8:**
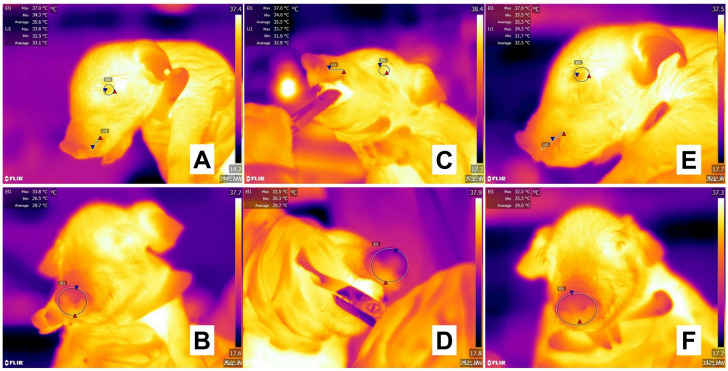
Evaluation of the thermal response in White large x Landrace piglets during teeth clipping. (**A**,**B**) Before teeth clipping in 1-day-old piglets, the thermal windows of the ocular surface (**A**, El1), upper lip (Li1), and nose (**B**, El1) show a maximum temperature (red triangle) of 37.0 °C, 33.6 °C, and 33.8 °C, respectively. (**C**,**D**) During the procedure performed with clippers, the ocular surface’s maximum temperature (red triangle) (**C**, El1) maintained the same value as basal recordings. However, the maximum temperature of the upper lip (Li1) increased by 0.1 °C and in the nasal window (**D**, El1) decreased by 0.3 °C. (**E**,**F**) After the routine procedure, although the maximum temperature of the ocular surface (**E**, El1) did not change compared to basal values, a progressive increase was observed in the upper lip (Li1) by 0.7 °C. The drop in the maximum temperature (red triangle) of the nasal region (**F**, El1) by 1.3 °C shows the physiological response of animals when perceiving potential painful stimuli. The thermal images were taken by the authors of the present review. Radiometric images were obtained using a T1020 FLIR thermal camera. Image resolution 1024 × 768; up to 3.1 MP with UltraMax. FLIR Systems, Inc. Wilsonville, OR, USA.

Teeth clipping is performed mainly in piglets to prevent damage to the sow’s udder or injuries to littermates, such as tail biting. Studies aimed at implementing IRT to evaluate this practice are limited. Radaelli et al. [120] used tooth temperature during teeth grinding in piglets 16 h after birth. At peak moments, the average tooth temperature ranged from 50 °C to 90 °C. These increases lasted 2 s. In spite of this rise, IRT showed that soft tissue remained unaffected when the procedure was performed correctly. Preliminary results of a study made by the authors during teeth clipping with clippers in piglets are shown in Figure 8. In this figure, the increase in the ocular surface temperature and the upper lip was recorded, while values in the nasal thermal window dropped by 1.3 °C due to the potential perception of pain and the peripheral vasomotor changes.

Since teeth clipping is usually performed to avoid lesions in littermates, some studies adopting IRT as a method to record the association between lesion score due to tail biting by conspecifics and changes in temperature due to inflammation and infection were studied by Teixeira et al. [121] in pigs. IRT at the tail base and ear base recorded lower temperatures in animals with lesion scores of 0–1 (between approximately 32 and 33 °C). However, considering some of the conflicting findings with respect to IRT presented, herein, it would be wise to corroborate this finding with other behavioral and physiological variables to reliably establish the piglets’ pain state welfare level before making any conclusions on the utility of IRT for monitoring in this procedure.

Other procedures that require analgesic protocols, such as laparotomy, have been studied by Viscardi et al. [122], who compared the analgesic effect of meloxicam and flunixin meglumine in sheep using pain-related facial expressions and IRT. The authors found no differences in facial expression scores or pain-associated behaviors assessed in animals treated with meloxicam and flunixin. Although no differences were observed between the temperature of the surgical site, both treatments reduced the temperature by 3.4 °C. The same has been reported in piglets, where the surface temperature of the cranial region and cortisol concentration decreased with the administration of meloxicam [112].

## 6. IRT as a Method to Monitor the Autonomic Response and Pain Intensity

A comprehensive evaluation of pain must consider the type of pain (acute and chronic) and its intensity (mild, moderate, and severe) [3,123]. Although current behavioral-based scales can be used to assess this, due to the multifactorial nature of pain and other factors such as masking of pain response in some species, the sensitivity of these tools is limited and often subjective [4,124]. For this reason, IRT has been suggested as a tool to aid in pain recognition [42]. Casas-Alvarado et al. [125] evaluated 21 bitches of different breeds undergoing ovariohysterectomy. After following an analgesic protocol of lidocaine alone or in combination with pure opioids, the thermal response of the lacrimal gland and eyelids did not show differences between treatments. Whilst this may suggest a lack of sensitivity of the IRT in determining pain intensity, given that the scores obtained on a validated pain assessment scale were also the same, a preferred interpretation may be that the analgesic effect of both drugs was similar.

**Figure 9:**
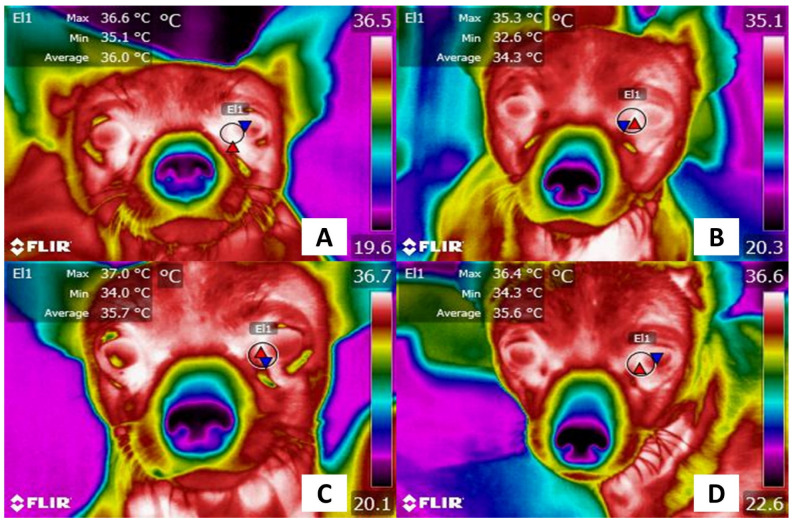
Thermal response associated with acute pain in dogs undergoing surgery. (**A**) Before surgery. A 1-year-old bitch of the Chihuahua breed was subjected to ovariohysterectomy under analgesic management with meloxicam (0.1 mg/kg IV). The maximum temperature of the lacrimal caruncle (El1) had values of 36.6 °C (red triangle), while the minimum was 35.1 °C (blue triangle). (**B**) 1 h after surgery. A decrease in the temperature of the lacrimal caruncle is observed, with maximum (red triangle) and minimum (blue triangle) values of 35.3 °C and 32.6 °C, respectively. This represents a decrease of 1.3 °C and 2.5 °C, respectively. This could represent a sympathetic response to the effect of pain despite the use of analgesics. (**C**) 2 h after surgery. After administering tramadol as rescue analgesia, the maximum and minimum temperatures of the lacrimal caruncle (El1) (37.0 °C and 34.0 °C) were recorded above the basal values, due to the effect of the drug that reduces sympathetic tone. (**D**) 3 h post-surgery. Although it is observed that the maximum temperature of the lacrimal caruncle (El1) decreased by 0.6 °C (red triangle), the minimum increased by 0.3 °C (blue triangle), which could be associated with post-surgical stability. The thermal images were taken by the authors of the present review. Radiometric images were obtained using a T1020 FLIR thermal camera. Image resolution 1024 × 768; up to 3.1 MP with UltraMax. FLIR Systems, Inc. Wilsonville, OR, USA.

During surgery or any procedure that causes acute pain, there is a need to evaluate analgesic treatments to ensure that the dose, selected drug, and protocol are adequate for the animals. IRT has the potential for use as part of this assessment. For example, pain management monitoring in a canine patient undergoing ovariohysterectomy is shown in Figure 9, where the intraoperative administration of intravenous meloxicam with rescue tramadol [126] restored the basal temperature of the patient in the lacrimal caruncle.

**Figure 10:**
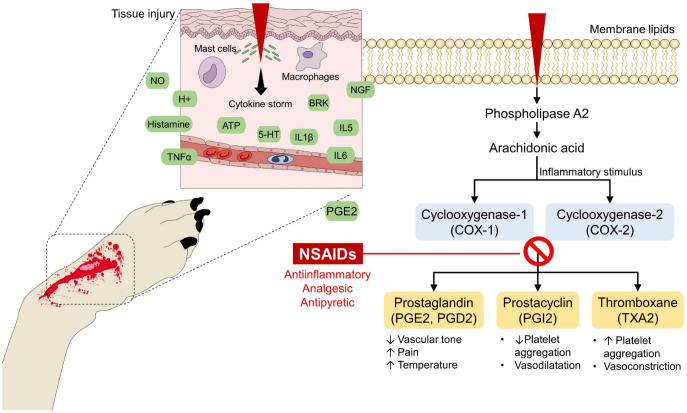
Mechanism of action and effect of analgesics in the nociceptive response and local inflammation. After tissue injury, the released pro-inflammatory mediators (e.g., NO, H+, histamine, 5-HT, among others) extend the local reaction to produce the five cardinal signs of inflammation. The administration of analgesic drugs, for example, NSAIDs, lessens this reaction by inhibiting the cyclooxygenase enzyme. In this way, reactions promoted by prostaglandin and prostacyclin, such as vasodilatation, pain, and local and systemic hyperthermia can be prevented. 5-HT: serotonin; ATP: adenosine triphosphate; BRK: bradykinin; H+: hydrogen; IL: interleukin; NGF: nerve growth factor; NO: nitric oxide; NSAIDs: non-steroidal anti-inflammatory drugs; PGE2: prostaglandin E2; TNFα: tumor necrosis factor alpha.

Tapper et al. [127] assessed pain intensity using pressure algometry and thermal sensitivity in 12 mixed-breed sows with lameness due to laminitis. They observed that the affected limbs tolerated less pressure and had a greater thermal response than healthy animals. Sodium salicylate and flunixin meglumine administration increased pressure tolerance and decreased the difference in thermal response between affected and healthy limbs. Thus, it was observed that analgesic treatment could reduce the radiation emitted due to an inflammatory event, as schematized in Figure 10 with the use of non-steroidal analgesics (NSAIDs). Likewise, Stubsjøen et al. [128] evaluated the infrared thermal response of the eye together with heart rate variability (HRV) to estimate the degree of pain in six sheep exposed to a forelimb tourniquet. The results showed that the stimulus decreased eye temperature by 0.47 °C, while heart rate and HRV increased. In another case report, an athlete-horse of the Amazon was diagnosed with septic arthritis, cursing with tachycardia (48 beats per minute) and tachypnea (60 breaths per minute) due to the perception of pain, by using IRT, ozone therapy applied to the injury site lowered by 1.9 °C the initial local temperature of 39.1 °C [129]. Accordingly, the discussed studies show that IRT can be considered a complementary tool to recognize acute pain and confirm the effectiveness of treatment.

Although there is a growing body of evidence suggesting that IRT may be useful as a pain assessment tool, it is clear from the above that there are conflicting findings. These conflicts may arise due to the measurements performed during research studies. The exclusive measurement of one parameter, such as maximum temperature, only provides limited data and cannot give an overall evaluation of the thermal state. Other elements such as thermal symmetry, chronology, and delta T, among others, are equally important for evaluation and contrast between studies. There is also controversy around the impact of various pharmacological agents on local temperatures and a lack of understanding as to the level of confounding this might create on results when trying to interpret in the context of pain. As an example, Holmes et al. [130] observed that local analgesics such as mepivacaine hydrochloride in distal joints in equine limbs did not change local temperature after administration. In contrast, reviews regarding the use of IRT argue that local analgesics such as lidocaine can cause hyperthermia due to the blockage of postganglionic fibers, which causes vasodilation and increased heat radiation [11,66]. As a result, further research is needed.

## 7. Future Research on IRT Application

Based on the research to date, it seems that IRT is beneficial as an additional method to recognize acute pain through the identification of superficial temperature changes under specific pathological conditions [7,131] such as tendonitis, laminitis, or mastitis [132,133]. However, it is necessary to establish the correlation between the thermal response and currently used tools to evaluate pain, such as pain scales [10] or behavioural-based measures of affective state [134]. Further, it is necessary to consider multiple measurements or features of the infra-red image to improve diagnostic accuracy, for example, symmetry and chronology. The utility of IRT is enhanced by increasing availability of complementary technologies, such as integration with parametric and non-parametric platforms, and the ability to use machine learning and artificial intelligence to automate data extraction and analysis.

Another aspect to be considered in future research is the correlation of autonomous activity through various tools such as IRT, infrared pupillometry, parasympathetic tone activity, heart rate variability, CARDEAn (cardiovascular depth analgesia), surgical plethysmography index (SPI), skin conductance, and photoplethysmography waveform amplitude (PPGA) [59]. For example, pupillometry has been applied to study the activation of the sympathetic nervous system after painful stimuli, modifying pupil diameter [135,136]. Regarding the parasympathetic tone activity index, its use with IRT could allow nociceptive monitoring of pain processes and their treatments by identifying hemodynamic instability, especially in patients under anesthesia [137,138].

**Figure 11:**
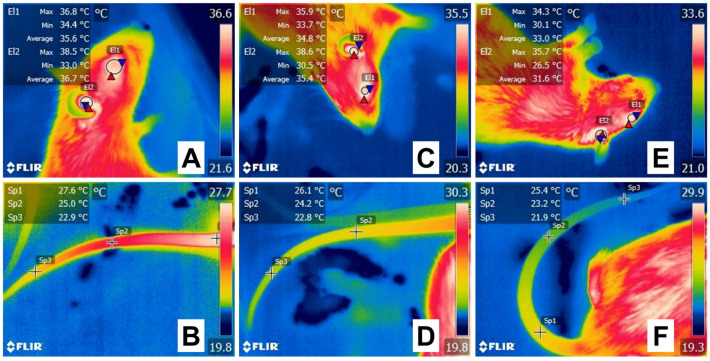
Thermal images in Wistar rats exposed to euthanasia with CO_2_. (**A**,**B**) Before the application of the euthanasia method, the ocular surface (El1), the auricular window (El2), and the base of the tail (Sp1) had a maximum temperature of 36.8 °C, 38.5 °C, and 27.6 °C, respectively. (**C**,**D**) During the euthanasia of the rodent with CO_2_ exposition inside a chamber, a general drop in the maximum temperature of the ocular surface (El1) and tail base (Sp1) by 0.9 °C and 1.5 °C was recorded. In contrast, an increase in the maximum temperature of the auricular region was reported (38.6 °C). (**E**,**F**) Within the first two minutes after exposition to CO_2_ euthanasia, all thermal windows showed a significant drop in temperature. When compared to basal values, the maximum temperature of the ocular region (El1) decreased by 2.5 °C, the auricular region (El2) by 2.8 °C, and the tail base (Sp1) by 2.2 °C. The progressive temperature drops in the rats, assessed by IRT, could help to determine the vasodilation effect of the euthanasia drug and could even be associated with pain when correlated with other evaluation tools. Maximal temperature is indicated with a red triangle and the minimal with a blue triangle. The thermal images were taken by the authors of the present review. Radiometric images were obtained using a T1020 FLIR thermal camera. Image resolution 1024 × 768; up to 3.1 MP with UltraMax. FLIR Systems, Inc. Wilsonville, OR, USA.

An important field of research where IRT could help to refine and promote animal welfare is during the slaughter or euthanasia of farm, companion, and laboratory animals in order to prevent pain and negative emotional states [74,139,140]. Weschenfelder et al. [74] have used infrared ocular thermography in pigs during slaughter to associate the physiological response with meat quality. In the case of animals used in science, preliminary results of the authors of the present article have shown that IRT can be applied during exposure to different types of euthanasia (injectable, inhalational, and physical) to determine the physiological reaction of the organism and potentially identify painful states. Figure 11 contains thermal images from Wistar rats during euthanasia with CO_2_.

**Figure 12:**
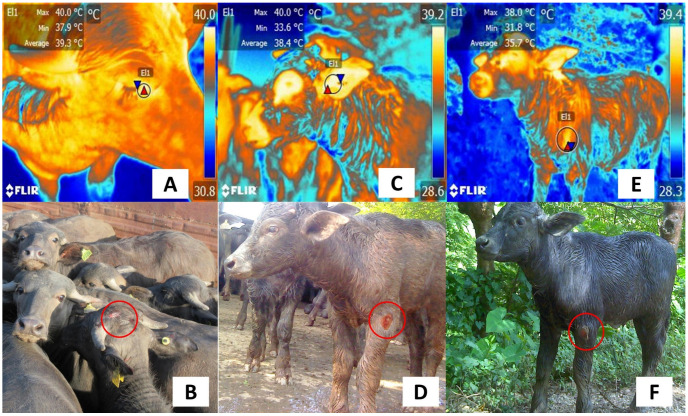
Recognition of inflammatory lesions in water buffaloes. (**A**,**B**) Thermal response due to lesion in the parietal region (red circle). An increase in the superficial temperature of the lacrimal caruncle (El1) can be associated with the lesion, with a maximum temperature of 40 °C (red triangle) and a minimum of 37.9 °C (blue triangle). (**C**,**D**) An ulcerative lesion on the elbow can be seen in the digital image. Through thermal imaging, the maximum temperature of the auricular region is 40 °C with a minimum of 33.6 °C. (**E**,**F**) The digital image shows a lesion in the shoulder region (red circle), whereas the radiometric image shows that the surface temperature of this region (El1) presented a maximum temperature of 38 °C (red triangle) and a minimum temperature of 31.8 °C (blue triangle), which would help to corroborate the presence of a lesion in the shoulder region as a result of the inflammatory process. The thermal images were taken by the authors of the present review.

The selection of thermal windows according to the species (e.g., farm [141], companion [66], laboratory [37], or wildlife under human care [7] is required to objectively categorize the changes in autonomic activity and the degree of pain. This could be applied during the transport of livestock, including water buffaloes, in whom IRT can detect changes in the surface temperature. This trait might have a repercussion on subproduct quality (Figure 12). Similarly, the application of IRT to detect subtle temperature changes in neonates [142,143,144], stress [141,145,146], or to assess the health status of the animals [147,148] must consider its use together with other techniques to improve the sensitivity and specificity of IRT as a complementary diagnostic method. Finally, it is important to consider that the current focus of IRT is to evaluate and monitor acute pain. There is controversy about the use of IRT to assess chronic pain or processes where inflammation is not present. Finally, IRT is increasingly being considered as a technology amenable to be used in precision farming as part of automated, computerized systems that help increase livestock production and allow early intervention to safeguard the welfare state [149,150].

## 8. Conclusions

Pain management in domestic animals has gained attention due to the increased recognition of its impact on welfare, production, and scientific model validity. Implementing tools in veterinary medicine to recognize pain, prevent said consequences, and establish proper anti-nociceptive and analgesic protocols is essential. As a non-invasive technique, IRT is an alternative for pain evaluation that has been used to detect acute processes during inflammatory states such as osteoarthritis or during routine surgical procedures such as castration, tail docking, and disbudding, where animals do not always receive analgesic drugs. According to the evidence, IRT applied to the ocular surface, the surgical wound, or the anatomical region where the inflammation occurs can identify temperature changes triggered by vasomotor changes in the dermis. IRT has helped to identify acute and chronic pain in companion and farm animals and has served as a complementary tool to evaluate the analgesic efficacy of different drugs.

At the current time it is sensible to use IRT with physiological parameters (e.g., heart rate, rectal temperature, heart rate variability), biomarkers (cortisol, substance P, and norepinephrine), activity levels (e.g., accelerometers), top-notch technologies (electroencephalography and electromyography), and behavioral-based scales to provide a comprehensive evaluation into the multidimensional components of pain.

## Data Availability

Not applicable.
